# A point of care sensor for detection of alcohols, aldehydes and esters in urinary metabolites of war veterans injured by sulfur mustard†

**DOI:** 10.1039/d4ra05461j

**Published:** 2024-09-19

**Authors:** Mohammad Mahdi Bordbar, Fatemeh Nobakht M. Gh., Azarmidokht Sheini, Maryam Alborz, Shahram Parvin, Mostafa Ghanei, Neslihan Kulahlioglu, Hosein Samadinia, Hasan Bagheri

**Affiliations:** a Chemical Injuries Research Center, Systems Biology and Poisonings Institute, Baqiyatallah University of Medical Sciences Tehran Iran h.bagheri@bmsu.ac.ir h.bagheri82@gmail.com; b Department of Chemistry, Technical and Vocational University (TVU) Tehran Iran; c Research and Development Department, Farin Behbood Tashkhis Ltd Tehran Iran; d Medical CBRN Defense Department, Institute of Defense Health Sciences, University of Health Sciences Gulhane Campus Keçiören Ankara Turkey

## Abstract

To discriminate between different alcoholic, aldehyde, and ester species of urine samples, a colorimetric sensor array consisting of dopamine-capped copper–silver bimetallic nanoparticles (Ag@Cu BMNPs) combined with 12 organic dyes is introduced. Based on the sensing mechanism, the nanozyme catalyzed the reactions of oxidation, dehydrogenation, and hydrolysis of volatile organic compounds. The products could alter the amount of hydronium ions in the detection media, making a variation in the color intensity of pH-sensitive indicators. Also, they could be connected to other organic dyes through nucleophilic/electrophilic or H-bonding interactions in order to form new complexes. The colorimetric responses of the sensor were visible to the naked eye and evaluated by image analysis software, thereby obtaining a unique detection pattern for each sample. The statistical data indicated that the sensor can completely distinguish between compounds with different functional groups. As a practical study, the efficiency of the sensor was investigated for the identification of the war veterans who injured by sulfur mustard in Iran–Iraq war and their differentiation from control people. Based on the output of the assay, the sensor was found to create a special color pattern for each studied group, achieving a total accuracy of 78.0% for this discrimination. The color change of the proposed sensor has a good correlation with the severity of the injury, being independent of the metabolic changes caused by the age of the participants. Accordingly, the fabricated sensor array can be a suitable tool to detect oxygen-containing compounds in environmental or biological samples.

## Abstract 2

To discriminate between different alcoholic, aldehyde, and ester species in urine samples of war veterans who exposed with sulfur mustard with healthy people, a paper-based colorimetric sensor array is introduced.

## Graphical abstract

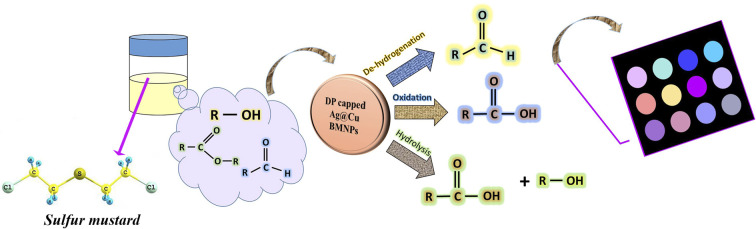

## Introduction

Metabolites, which are small molecules constituting the metabolic products of living cells, are capable of stimulating, catalytic, and inhibitory effects.^1^ These compounds mainly have hydroxyl, carbonyl, amine, and epoxy functional groups, which can be utilized as disease diagnostic markers.^2^ The concentration of oxygen-containing compounds, including alcohols, aldehydes, ketones, esters, amides, ethers, and acids, has been observed to vary with regard to a diverse range of diseases. These diseases encompass cardiovascular disorders, neurological disorders, lung cancer, leukemia, stomach cancer, kidney disease, diabetes, urinary system infections, and viral infections.^3,4^ Since the quantity of other compounds increases, the concentration of certain species decreases as the body's defense mechanism progress.^5^ The chromatographic (particularly gas chromatographic) and spectrometric (*e.g.*, nuclear resonance) diagrams of these compounds exhibit distinctive peaks, which enable precise quantification of the presence and quantity of a particular chemical species.^6–9^ However, in order to transport a specific volume of the sample containing the analyte to the laboratory, complex equipment and an operator are required to perform the analysis, which is a high-cost task. In fact, not only is the user unable to afford the implementation of this method, but so are certain medical laboratories. Furthermore, the setting up of the instrument and the preparation of the sample in advance lengthen the analysis process.^10^ Cost and time savings in analysis can be achieved through the development of miniature instruments. In recent times, there has been a utilization of electronic nose and tongue-based measurement devices for the assessment of metabolites. These devices offer certain benefits, including the ability to generate a unique pattern with high accuracy for oxygen-containing compounds possessing diverse functional groups. Additionally, they can detect particular substitutions in materials with varying structures, such as the presence of arene ring and nitro groups, as well as an increase in the alkane chain. Moreover, these devices can effectively determine volatile compounds with a low concentration of 10 ng mL^−1^ by applying nanoparticles (NPs) as sensing elements.^11–13^ The utilization of nanomaterials enhances the performance of array-based sensors owing to their distinctive characteristics, such as the surface plasmon resonance or high surface-to-volume ratio.^14^ Moreover, the surface modification of NPs by coating agents with various structures makes them participate in diverse interactions, including nucleophilic, electrostatic, H-bonding, covalent, and hydrophobic interactions.^15,16^ By possessing enzyme-like properties, some NPs are called nanozymes.^17^ In contrast to natural enzymes, these more stable compounds can be mass-produced inexpensively and with the capacity to modify their catalytic activity.^18^ Depending on the intrinsic properties of nanozymes, the compounds can imitate the catalytic activity of oxidoreductase, hydrolase, or lyase.^19^ The product of the enzymatic reaction is identified and quantified by an electrochemical or a colorimetric method. For example, glucose is oxidized in accelerated reaction when glucose oxidase is present in the reaction media, producing hydrogen peroxide, causing the 3,3′,5,5′-tetramethylbenzidine turned to oxidized form in the presence of nanozyme (such as Ag nanozyme synthesized by silk sericin) and a change in its color.^20^ On the other hand, MoS_2_ quantum dots are capable of catalyzing the oxidation reactions of oxygen-functionalized volatile compounds, so that one can detect the acidic product using pH-sensitive dyes.^21^

The activity of nanozymes as a catalyst is influenced by a different factors, including the composition, morphology and size of the synthesized NPs. Additionally, environmental variables including pH, temperature, and light can also affect the surface modification of the NPs.^22^ While monometallic nanoparticles (NPs) such as silver, gold, copper, platinum, and palladium possess advantageous catalytic capabilities, the utilization of composite NPs comprising two or more metals enhances the catalytic efficiency of nanozymes in facilitating chemical reactions. While monometallic NPs (*e.g.*, palladium, silver, gold, copper, and platinum) possess advantageous catalytic characteristics, the capability of nanozyme to catalyze a chemical reaction is enhanced when a composite NP containing two or more metals is utilized.^23^ It is even possible to regulate the activity of the nanozyme by changing the ratio of metals. The higher performance of the polymetallic NPs is due to their intrinsic properties, arising from the synergy of two or more metals in addition to the physiochemical characteristics of monometallic NPs.^24^ Alternatively, modifying the nanozyme surface with coating agents leads to its stability against aggregation, while also increasing the nanozyme catalytic activity. The coating agents containing amino, carboxyl or sulfhydryl groups may have a better effect on the performance of nanozymes.^25,26^

The term “chemical injury” generally refers to a condition where humans are exposed to toxic chemical compounds such as sulfur mustard, resulting in eye, skin, gastrointestinal tract, and respiratory tract damage.^27^ The best method for diagnosing and monitoring the severity of the chemical injury is lung biopsy, which is a costly, time-consuming, and invasive method that the patient prefers to refuse.^28^ Since concentration profiles of metabolites are different in the body fluids of injured and control individuals,^29^ the sulfur mustard injury can be detected non-invasively by employing a sensing device to monitor the compounds acid, aldehyde, ketone, amine, ester, and alcohol in a biological sample (*e.g.*, urine). Our research group tried to develop a suitable sensor for rapid and simple detection of exposure to hazardous materials. As a result, a pocket sensor was fabricated for identification of war veterans injured by the sulfur mustard through examining the changes in the type and concentration of plasma sample metabolites in comparison with the patterns of healthy people.^30^ However, plasma collection is an invasive and time-consuming. To solve this problem, the proposed sensing device was used to monitor a set of index metabolites in urine samples.

The sensing device contains a copper–silver bimetallic nanozyme functionalized with dopamine same as our previous study.^30^ This nanozyme is expected to catalyze the oxidation reactions of aldehydes, the dehydrogenation of alcohols, and the hydrolysis of esters, resulting in acidic, aldehyde, and alcoholic products. The second component of the sensing element is an organic dye that responds to variations of the hydronium ion concentration in the reaction medium or that creates a new complex with the enzymatic reaction products. In the proposed sensing device, the nanozyme is mixed with 12 organic chemical species with different structures,^30^ providing a sensor array based on an electronic nose. This array is designed on a common filter paper substrate. The performance of the fabricated sensor is evaluated in two parts: first, the efficiency of the sensor is evaluated to identify and discriminate chemical materials related to alcohol, aldehyde and ester groups; and second, the practical ability of sensor is studied for tracking these metabolites in urine samples in order to diagnose the individuals (war veterans) affected by the sulfur mustard.

## Results and discussion

The use of hybrid sensors made of two or more sensing elements with different physicochemical properties can increase the efficiency of an assay like cut-off and selectivity when identifying the materials of the same groups with similar structures.^30,31^ In this study, 12 organic dyes were separately dissolved in Ag@Cu BMNPs coated with dopamine in order to fabricate an array of sensors on a paper substrate. This array was exposed to volatile compounds with alcohol, aldehyde, and ester structures. The NPs catalyzed the reaction of dehydrogenation of alcohols, oxidation of aldehydes, and hydrolysis of esters. The products had Brønsted acid–base, Lewis acid–base, or H-bonding interactions with the organic dyes. Conversely, the aforementioned measurement procedure was employed to assess the disparities in volatile compound emissions from urine samples collected from sulfur mustard-injured and healthy subjects. More details about the results of the proposed assay are presented in the following sections.

### Characteristics of the synthesized Cu@Ag BMNPs

The characterization of the synthesized Cu@Ag BMNPs including UV-Vis spectrum, the surface electrical charge, average dynamic size by dynamic light scattering (DLS), mapping and EDS spectrum by scanning electron microscopy has been previously reported in our published paper.^30^

### Analysis of volatile organic compounds

#### Optimization of conditions

Among the volatile organic compounds to which the proposed sensor was subjected were acetaldehyde, ethanol, butanol, hexanol, butyraldehyde, hexanaldehyde, ethyl acetate, ethyl butyrate, and ethyl hexanoate. For this purpose, the container which was included a paper based sensor, was filled with 10.0 mL of pure material.

Once the reaction container was sealed, the reaction of the volatile compounds and the sensing receptors was enforced. The result of the proposed assay can be influenced by some parameters, such as the amounts of the color dye dissolved in the NP solution and the interaction time between the analyte and sensing elements, which must be optimized. In this section, the optimization aimed to achieve the conditions for separating the studied volatile compounds and their families with high total accuracy. Therefore, the best conditions were selected by using the discriminant ability function (DAF). Basically, DAF calculates the ratio of between-group variance to within-group variance, and its highest value appears at the optimal point of each parameter.^32^

In order to optimize the concentration of color indicators, five different amounts of these compounds were dissolved in the synthesized NPs, thus preparing solutions with concentrations ranging from 0.2 to 1.0 mg mL^−1^. It should be noted that the concentration of 12 color indicators was the same in each array. The experiment was executed, and the DAF values were computed for each concentration, as illustrated in Fig. S1a.† It is evident that the best discrimination among the studied analytes is observed when the colored solutions have a concentration equal to 0.4 mg mL^−1^ are used in the sensor fabrication. Note that the lower amounts of dyes do not have enough interaction sites. On the other hand, the color intensity of the concentrated solutions prevents to observe the variation in the sensor responses after exposing to analyte.

In another experiment, the fabricated sensor was exposed to the studied targets for a period of 90 min. The experiment was continued at ambient temperature, and the response of the sensor was recorded at a time interval of 15 min. The DAF values depicted in Fig. S1b† show that the assay responses are almost constant after 60 min, indicating that the interaction of the analyte and the sensing receptors reaches equilibrium. Then, for collecting the sensor responses in subsequent studies, this time was selected.

#### Investigation of color changes

**Fig. 1:**
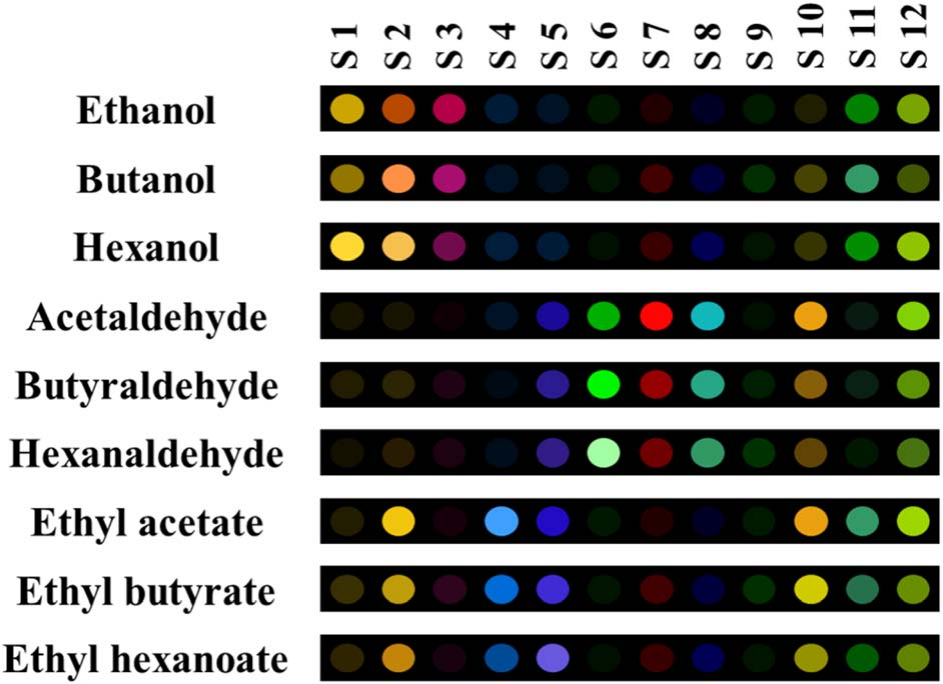
The colorimetric patterns for nine volatile compounds. The analysis was performed under hood at 25 °C for 60 min.

The synthesized BMNPs were combined with 12 color indicators with different structures and properties. Basically, organic dyes either have specific functional groups that can chelate with the nanozyme reaction product or change color due to the variation in the hydronium ion concentration in the reaction medium. Fig. 1 illustrates the response of the constructed array to the nine analytes that were investigated. As observed, all the sensing elements reacted to the volatile compounds, although the intensity of the interactions was different. To illustrate, the presence of alcohol compounds induces distinct color alterations in nanozymes conjugated with ARS, IC, TB, and EBT dyes. Conversely, the sensing elements comprising CR, PR, PAR, BPR, and MU exhibit a more robust interaction with aldehyde materials. The reaction product of the dehydrogenation of alcohols can make nucleophilic- or H-bonding with the dyes in the first category. Moreover, the compound obtained from the oxidation of aldehydes was an acid, thereby changing the pH and the color of the dyes in the second category. On the contrary, esters have a tendency to interact with sensor components that comprise IC, MG, CR, MU, and CAS. As previously stated, the color pattern recorded for esters exhibited similar characteristics to the patterns previously identified for alcohols and aldehydes. S12, which consisted of BMNPs integrated with EBT, exhibited a strong tendency to detect all volatile compounds.

As illustrated in Fig. 1, some sensors were more selective for a specified target. As an example, S1 (NPs integrated with ARS) and S3 (NPs integrated with TB) interacted with alcohols, S4 (NPs integrated with MG) responded to esters, and the three sensors consisting of S6 (NPs integrated with PR), S7 (NPs integrated with PAR), and S8 (NPs integrated with BPR) had high selectivity to aldehydes. The intensity of the color changes of some sensing elements depends on the number of carbon atoms in the studied analyses chemical structure. Notably, enhancing in the number of carbon atoms in the aldehyde compounds illuminates the color of the sensing element S6, and decreases the color intensities of the sensing receptors defined as S7 and S8.

To evaluate the stability of sensor responses, a specified number of paper based sensors were made under the same conditions. These sensors were exposed to three compounds of ethanol, acetaldehyde and ethyl acetate immediately and also during 100 days (within a 15 days interval) after sensor fabrication. The experiment was repeated for three times in a day and the sensor response was collected as Euclidean norm. As indicated in Fig. S2,† the validity of the sensors for detecting the studied volatile compounds is 75 days. After that, the initial color of the sensor changes due to physicochemical variations.

#### Discrimination ability

**Fig. 2:**
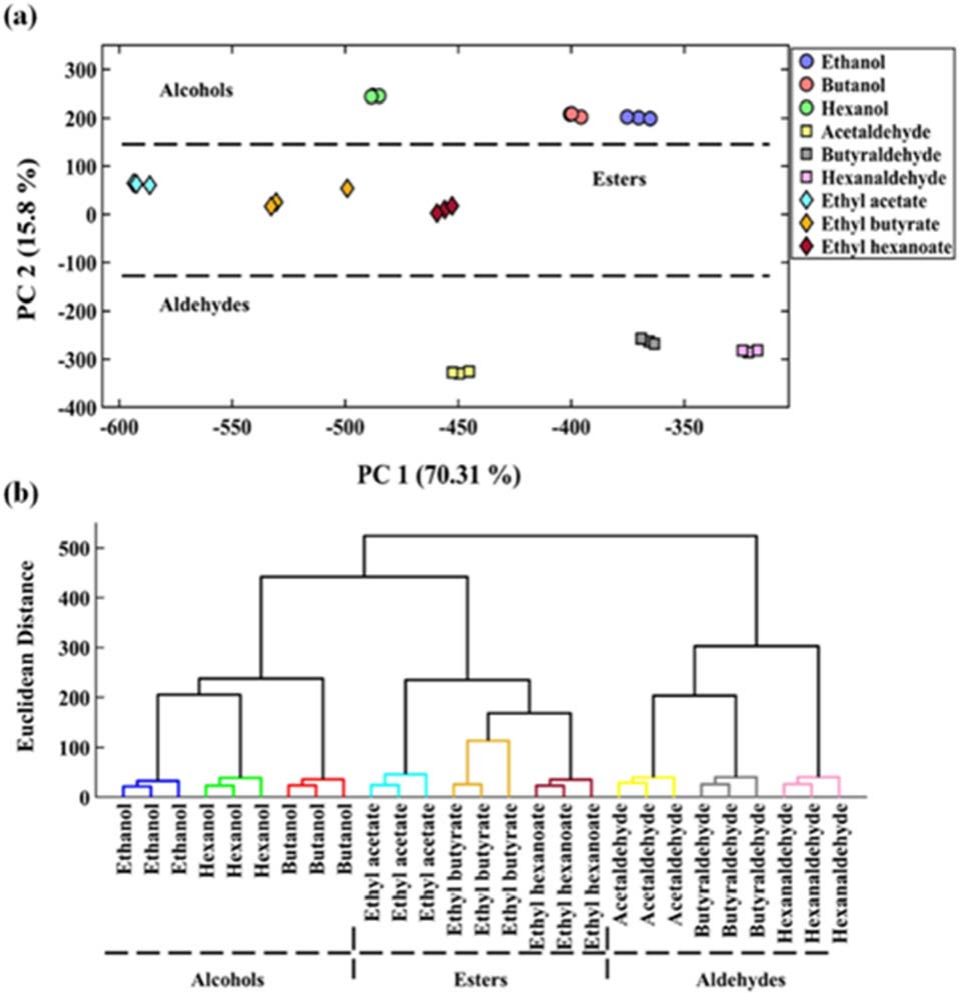
The discrimination results provided by (a) PCA and (b) HCA.

In order to verify the sensor array's performance to distinguish among the volatile compounds studied, the principal component analysis (PCA) and hierarchical cluster analysis (HCA) methods were implemented. For this purpose, the response vectors obtained from the image analysis were compiled into a matrix with a size of 27 × 36, and inserted into the multivariate pattern recognition methods as input data. The PCA score plot is presented in Fig. 2a. This indicates that the volatile compounds are dispersed within the range of two first principal components, which account for 86% of the variances that were explained. According to this figure, not only the studied analytes are well separated, but also each sample is correctly divided into the three groups of alcohols, aldehydes, and esters. Interestingly, the extracted pattern for esters is placed between the distribution of data points for alcohols and aldehydes, being consistent with the visual data obtained by the colorimetric sensor (Fig. 1). Alternatively, the discrimination between the volatile compounds was investigated using HCA, resulting in providing the dendrograms shown in Fig. 2b. In this case, the analytes are grouped in their own related cluster, and the classes of alcohols, esters and aldehydes are categorized with total accuracy of 100%.

#### Diagnosis of injury caused by sulfur mustard

In previous section, the ability of the sensor was evaluated with respect to the identification and differentiation of volatile chemical compounds. The presence of these compounds within biological samples or alterations in their concentration can be used as a marker in the diagnostic process of a disease.^30^ This section is aimed to track the reaction of a non-invasive sensor to volatile metabolites found in urine samples, with the objective of detecting sulfur mustard injury.

#### Optimization

Due to the diminished volatility and concentration of metabolites in complex urine samples relative to pure volatile compounds, it is possible that the sensor may not exhibit a sufficient response to discern metabolite variations between control and injured persons. Hence, it was necessary that one optimize the characteristics that impact the sensor's performance, including temperature and time.

Aiming for the studied groups to be sufficiently differentiated, the optimal conditions were chosen. In this regard, the sensor array was exposed to five samples (from each group) with three replicates, and the sensor response was subsequently determined by calculating the Euclidean norm. Finally, the DAF equation was utilized to find the optimal value of each parameter. To start, the sensor was subjected to a one-hour exposure to the volatile metabolites present in the urine sample within the temperature range of 25–95 °C. The sample was transferred to an oven when temperatures above 25 °C. The data given in Fig. S3a† shows that more metabolites were accumulated in the headspace of the reaction container when increasing the temperature, thus clarifying the difference between the metabolite profiles of urine samples for the injured and control individuals. Therefore, considerable discrimination occurs between the two studied groups. However, beyond the temperature of 75 °C, the DAF value is constant. This temperature was selected to collect the sensor responses for subsequent studies.

In the next step, the interaction between the sensor and the urine components was investigated during 0–150 min. As illustrated in Fig. S3b,† it is found that the time required for the release of the volatile compounds in the urine sample, and their penetration into the sensor texture and complete interaction with the sensing elements is 90 min. After that, no noticeable changes in the detection elements were observed. Accordingly, the result of assay was gathered after 90 min for further studies.

#### Sensor response

**Fig. 3:**
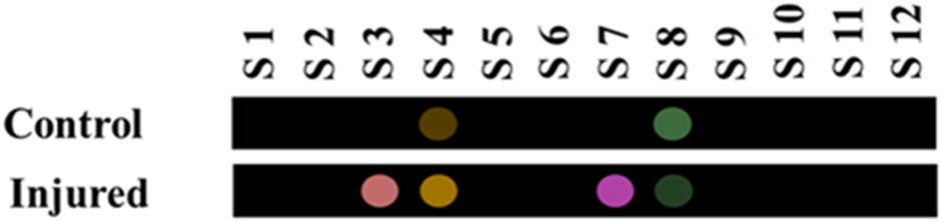
The response of sensor for analysis of urine samples of control and injured individuals. The analysis was performed in oven at 75 °C for 90 min.

Following the optimization of the experiment conditions, a volume of 10.0 mL of urine sample was poured into the reaction container, where its volatile compounds were allowed to be exposed to the proposed sensor array. Under similar conditions, the array response to water vapor and air (in the empty container) was obtained. The interaction results were calculated based on eqn (1)–(6) (see Experimental section). Fig. 3 illustrates the color difference pattern observed in the samples obtained from both the control group and the injured individuals. As observed, the response of sensing elements S4 (nanozyme + MG) and S8 (nanozyme + BPR) changed in the presence of both the studied samples. The sensors exhibited a response to prevalent ester and aldehyde compounds present in the urine samples. Nevertheless, the concentration of these compounds may decrease or increase, leading to a decrease or an increase in the color intensity obtained for the sensing elements. As shown in Fig. 3, elements S3 (nanozyme + TB) and S7 (nanozyme + PAR) exhibited a response exclusively to the components present in the samples of the individuals who had sustained a sulfur mustard injury.

Upon further examination, it was found that the response (Euclidean norm) of the sensing elements S3 and S7 has a linear relationship with the severity of the injury, as indicated by their respective correlation coefficients (*R*^2^) of 0.928 and 0.959 (Fig. S4†). These plots can be utilized to derive an approximation of the severity of the injury that sulfur mustard inflicts upon an unidentified person.

**Fig. 4:**
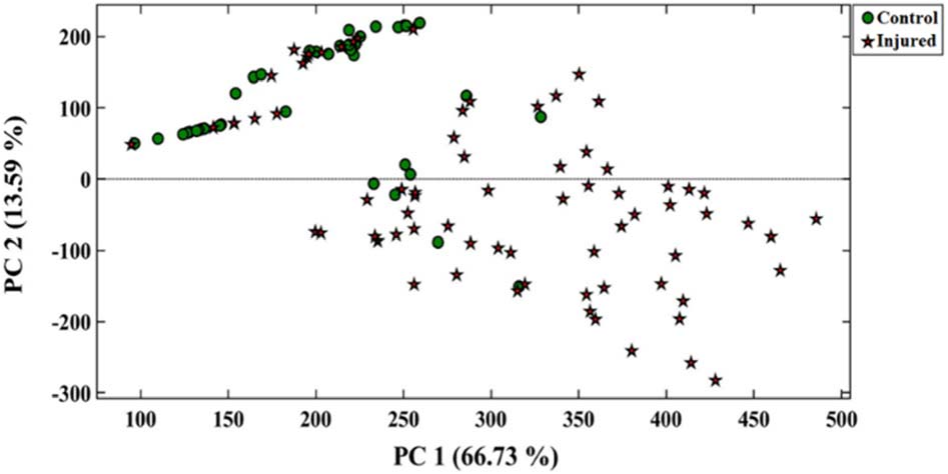
The PCA score plot for discrimination of control and injured individuals.

The performance of the designed array was evaluated using the PCA–LDA method. Fig. 4 displays the pattern of the data scattered between the first two principal components, comprising 84.53% of the total variance. As illustrated in Table 1, the sensor exhibited a 78.0% accuracy rate in differentiating between the control and injured groups. The sensitivity obtained for the correct diagnosis of injured and healthy individuals was calculated to be 79.0% and 78.0%, respectively. Of course, the most injured people who were misclassified have mild injuries. It seems that the metabolic changes caused by sulfur mustard injury in these people were so low that they could not be detected by the proposed assay.

**Table 1:** The statistical parameters obtained by PCA–LDA

Accuracy (%): 78.0, error rate (%): 22.0		
	Control	Injured
Sensitivity (%)	78.0	79.0
Specificity (%)	79.0	78.0

#### The effect of volunteer's age

To examine how the age of the volunteers affected the sensor's response to metabolic changes, the correlation between the age of each participant and the Euclidean norms computed for the urine sample was assessed. The Pearson correlation coefficient for the control and injured groups was equal to −0.306 and −0.099, respectively. The respective *p*-values were determined as 0.106 and 0.409. Based on this information, there was no acceptable and significant correlation between the array responses and the volunteer's age.

#### Reproducibility

Four different sensor arrays were prepared under the same conditions and exposed to urine samples from injured and healthy participants in order to investigate the reproducibility of the assay signals. Following the acquisition of the sensor response and the computation of the Euclidean norm, the relative standard deviation (RSD) was determined for the aforementioned four measurements, as illustrated in Fig. S5.† The RSD values were equal to 4.67% and 5.62% for samples of the injured and control individuals, respectively. The low relative errors approve the reproducibility of the variation in the response of the sensor array after exposing to the volatile compounds in urine samples of both studied groups.

## Experimental section

### Materials

Copper(ii) nitrate trihydrate(Cu(NO_3_)_2_·3H_2_O), silver nitrate (AgNO_3_), sodium borohydride (NaBH_4_), ethanol, butanol, hexanol, acetaldehyde, butyraldehyde, hexane aldehyde, ethyl acetate, ethyl butyrate, and ethyl hexanoate were provided from Merck Company. Dopamine (DP) and indicators such as Alizarin Red S (ARS), indigo carmine (IC), toluidine blue (TB), malachite green (MG), Congo red (CR), phenol red (PR), pararosaniline (PAR), bromopyrogallol red (BPR), erythrosine (ER), murexide (MU), Chrome Azurol S (CAS), and Eriochrome Black T (EBT) were purchased from Sigma Aldrich. Whatman paper (grade 2) was used to create the paper-based sensor.

### Equipment and software

The shape of sensor was created in AutoCAD 2016 environment. The prepared design was printed on the Whatman paper with the help of a printer (such as HP LaserJet, Model 1200). The sensor images were captured and evaluated by a scanner device (Model CanoScan LiDE 220) and ImageJ software (version 1.51n, National Institutes of Health), respectively. To implement the multivariate pattern recognition methods and other statistical analyses, MATLAB (version R2021) and SPSS (version 22, Chicago, IL, USA) were utilized.

### Synthesis of bimetallic nanoparticles (BMNPs)

The synthesis procedure for bimetallic NPs (BMNPs) was similar to the previous reports,^33^ although with some modification processes. First, aqueous solutions of Cu(NO_3_)_2_·3H_2_O, AgNO_3_, and dopamine were prepared separately and its concentration was adjusted at 0.1 mol L^−1^. A fresh NaBH_4_ solution (0.1 mg mL^−1^) was also prepared. Next, 50.0 μL of copper ion solution and 50.0 μL of dopamine solution were added to 100.0 mL of deionized water under stirring. After 5 min, the resulting solution was mixed with NaBH_4_ solution, and remained under stirring for 15 min. In the next step, silver ions solution with the volume of 50.0 μL was injected to the container, and the reaction continued for 20 min. Finally, a yellow-brown solution was formed, thereby confirming the synthesis of Ag@Cu BMNPs.

### Preparation of sensing elements

In order to prepare the sensing elements, the optimal amount of each organic dye was accurately weighed and dissolved in 1.0 mL of the synthesized BMNPs.

### The sensor fabrication process

**Fig. 5:**
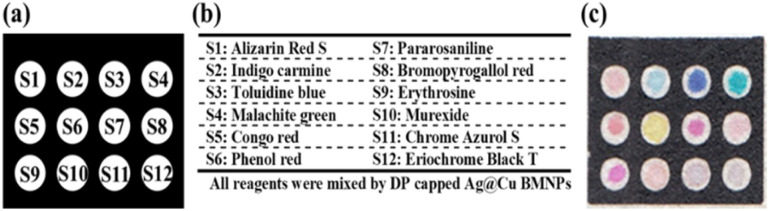
The structure of sensing device: (a) the designed pattern, (b) the name of sensing element and (c) the fabricated sensor.

The sensor pattern comprised 12 small white circles and was represented by a black rectangle measuring 1.0 cm by 0.75 cm. This scheme was drawn by AutoCAD software (Fig. 5a) and plotted on paper with the help of a printer. The paper was warmed gradually at 200 °C. Forty five minutes was required that the black region of the paper became hydrophobic.^34^ Moreover, 0.3 μL of each sensing element was added to the small circles based on the list shown in Fig. 5b. The created sensor can be observed in Fig. 5c.

### The practical method

A schematic for the experimental method is provided in Fig. S6.† As illustrated, the designed sensor (Fig. S6a†) was pasted on a plastic substrate (Fig. S6b and c†). The second side of the substrate had a double-sided adhesive tape, which allowed the sensor to be attached to the cap of the reaction container (Fig. S6d†). The glass container (150 mL) included alcoholic (ethanol, butanol, and hexanol), aldehyde (acetaldehyde, butyraldehyde, and hexanaldehyde) or ester (ethyl acetate, ethyl butyrate, and ethyl hexanoate) chemical compounds (Fig. S6e†). The sensor was exposed to these volatile compounds under the hood at a temperature of 25 °C for a certain period of time (Fig. S6e†). A scanner was utilized to record the alteration in color of the sensing elements that occurred during the interaction between the chemical vapors and the sensor (Fig. S6g†). The ImageJ software was applied to calculate the variations of sensor colors. For this purpose, the color difference of each sensing element was determined prior to and subsequent to the interaction (Fig. S6h†). The result was presented as three values in RGB color space (Fig. S6i†). In another experiment, the response of the sensor to an empty container (*i.e.*, without a chemical compound) was investigated. In essence, the ultimate RGB values for each sensing element were calculated by subtracting the values acquired by the sensor under the chemical substance's presence and absence, as detailed below:

1 *R* = Δ*R*_Full container_ − Δ*R*_Empty container_

2 *G* = Δ*G*_Full container_ − Δ*G*_Empty container_

3 *B* = Δ*B*_Full container_ − Δ*B*_Empty container_

The preceding equation provides the changes in the color of each sensing element prior to and subsequent to its interaction with the analyte, denoted by Δ*R*, Δ*G*, and Δ*B* (the empty or full container). These analyses were performed for 12 sensing elements individually, resulting in a vector containing 36 numerical values (12 sensing elements × 3 color elements) for a sensor array.

### Urine sample analysis

#### Selection of participants

Iran, as the largest victim of the use of prohibited chemical weapons, currently has over 60 000 veterans from the Iran–Iraq war suffering from chemical warfare injuries who require ongoing medical care. This research was conducted at Baqiyatallah-Azam Hospital in the years of 2021–2023. The individuals as war veterans who were injured by sulfur mustard in the Iran–Iraq war (from 1980 to 1988) were invited to participate in this study. These volunteers (114 war veterans) with confirmed the chemical injuries based on official documents, documented past medical history and an examination by the subspecialist have been included in this study. The purpose of the study and the experimental procedures were explained to the participants, and they were asked to fill out and sign the informed consent and questionnaire carefully. The control subjects were selected from patients admitted to different clinical departments of the hospital. These subjects (36 persons) did not have any disease that mentioned as the exclusion criteria.^30^ In order to differentiate the control group from the injured group and ascertain the extent of the injury, airflow measurement was performed utilizing spirometry, the prevailing technique for diagnosing obstructive pulmonary disease.^35^

To perform spirometry, inspiratory and expiratory flow rates were plotted as a flow-volume loop. Finally, the ratio of forced expiratory volume in the first second (FEV1) to forced vital capacity (FVC) was calculated.^36^ In fact, the fraction of the vital capacity that can be expelled from the lungs in the first second during exhalation was measured. According to this method, control subjects had FEV1 > 80 (FEV1/FVC > 70% predicted). The FEV1 values for the person with a mild injury are more than 80% but the amount of FEV1/FVC is lower than 70%. For other injured people, the values of FEV1 are located in the range of 50–80 for moderate and 30–50 for severe injuries.^37^

#### The detection method

The mechanism of urine samples analysis was similar to that presented in the previous section, except the reaction container was filled with 10 mL of urine sample instead of pure organic solvents (Fig. S6f†). The sensor was exposed to vapors emitted from the urine sample at a specified temperature and the result was gathered after a certain period of time (Fig. S6f†). The color responses obtained from the scanner subsequent to the reaction were utilized to calculate the numerical value of each sensing element through the image analysis software. Given that a considerable proportion of the urine sample consisted of water, the sensor's responses to the water vapor were computed. The changes of the sensor signal due to the content of the empty container were also tracked. Finally, the colorimetric signal of each sensing element was determined from the following equations:

4 *R* = Δ*R*_Urine sample_ − Δ*R*_Water vapor_ − Δ*R*_Empty container_

5 *G* = Δ*G*_Urine sample_ − Δ*G*_Water vapor_ − Δ*G*_Empty container_

6 *B* = Δ*B*_Urine sample_ − Δ*B*_Water vapor_ − Δ*B*_Empty container_

where the values of Δ*R*, Δ*G*, and Δ*B* represent the color changes of each sensing element before and after interaction with the contents of the reaction container. The final response for each urine sample was a 36-member vector, which was used for subsequent studies.

#### Statistical calculations

The Euclidean length was used to determine the total result of assay as a unique number, according to eqn (7):7
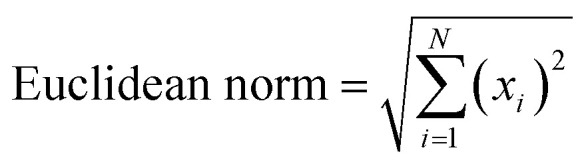
where *x*_*i*_ is the *i*th number of the data vector obtained for the sample.

For discriminating of the studied species (volatile organic compounds and urine samples), multivariate pattern recognition methods such as principal component analysis–linear discriminant analysis (PCA–LDA), and hierarchical cluster analysis (HCA) were used. The relationship of parameters such as the disease severity and age with the sensor response was evaluated using Pearson's correlation coefficient with a specific *p*-value.

## Conclusions

A colorimetric sensor has been designed consisting of two components: BMNPs and color indicators, allowing for the detection of alcohol, aldehyde, and ester volatile compounds through an enzymatic process. This sensor was able to discriminate not only all the compounds in a certain category but also the three studied groups. The efficiency of the proposed sensor was investigated as a non-invasive method to differentiate between war veterans with sulfur mustard injured and healthy individuals by analyzing volatile compounds in their urine samples. Based on the obtained data, this sensor could detect war veterans who chemically injured by sulfur mustard. Besides, by tracking the response intensity of the sensor array, the severity (mild, moderate, and severe) of the injury was determined. The sensor responses were highly reproducible, and the parameters, such as the individual's age, did not affect the color changes of the sensing elements. Significant features such as miniature size, low-cost fabrication and simple design, in addition to acceptable sensitivity and specificity, non-invasiveness, availability, and user-friendliness can introduce this method as a reasonable alternative to the current cost-effective standard methods. Additionally, this sensor can be used as a complementary test alongside our previously developed sensor for analyzing plasma metabolites of the war veterans with sulfur mustard injury.^30^ As a limitation for this method, the sensing device is not reversible and can only be used as a disposable sensor.

## Ethical statement

The research ethics committee of Baqiyatallah University of Medical Sciences has approved the project (Approval ID: IR.BMSU.BLC.1400.006).

## Data availability

The data that support the findings of this study are available on request.

## Conflicts of interest

There are no conflicts to declare.

## Supplementary Material

RA-014-D4RA05461J-s001
