# Supplementary material for: A point of care sensor for detection of alcohols, aldehydes and esters in urinary metabolites of war veterans injured by sulfur mustard
Source: RSC Adv. 2024 Sep 19;14(40):29518–25. doi: 10.1039/d4ra05461j (PMC11411418; doi:10.1039/d4ra05461j)
Supplement: RA-014-D4RA05461J-s001 [file RA-014-D4RA05461J-s001.pdf]

Supplementary Information (SI) for RSC Advances.
This journal is © The Royal Society of Chemistry 2024

Supporting information

for

A point of care sensor for detection of alcohols, aldehydes and esters in urinary metabolites of war veterans injured by sulfur mustard

Mohammad Mahdi Bordbar[1], Fatemeh Nobakht M. Gh [1], Azarmidokht Sheini[2], Maryam Alborz [3], Shahram Parvin [1], Mostafa Ghanei [1], Neslihan Kulahlioglu [4], Hosein Samadinia [1], Hasan Bagheri[1,]*

[1] Chemical Injuries Research Center, Systems Biology and Poisonings Institute, Baqiyatallah University of Medical Sciences, Tehran, Iran

[2] Department of Chemistry, Technical and Vocational University (TVU), Tehran, Iran

[3] Research and Development Department, Farin Behbood Tashkhis LTD, Tehran, Iran

[4] University of Health Sciences, Institute of Defense Health Sciences , Medical CBRN Defense Department Gulhane Campus, Kecioren/ANKARA, Turkey

Corresponding author: h.bagheri@bmsu.ac.ir; h.bagheri82@gmail.com

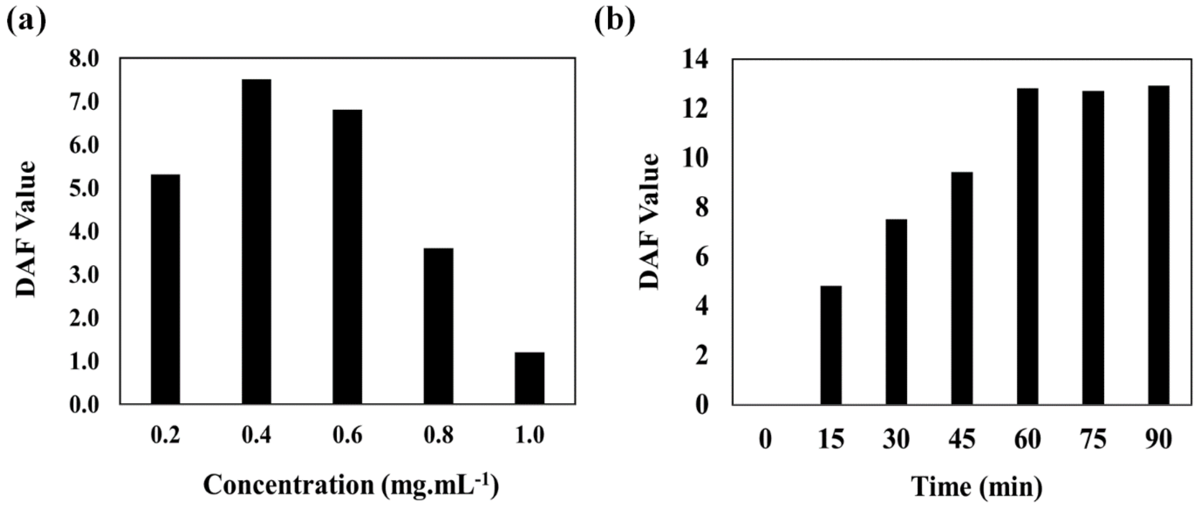

Figure S1. The optimization of effective parameters for analysis of pure compounds: (a) concentration of organic dyes and (b) reaction time.

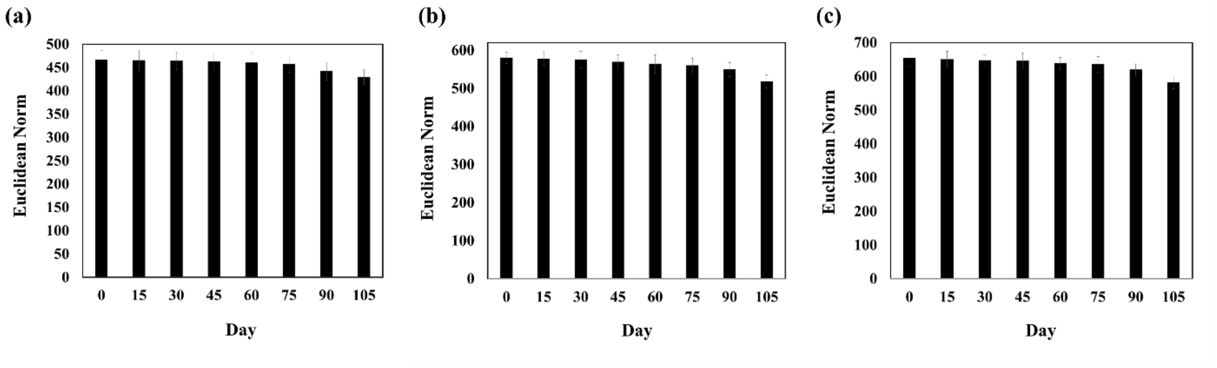

Figure S2. Evaluation of the Euclidean norms of sensor for detection of (a) ethanol, (b) acetaldehyde and (c) ethyl acetate. Sensor responses were collected immediately and also during 100 days (within a 15-day interval) after sensor fabrication.

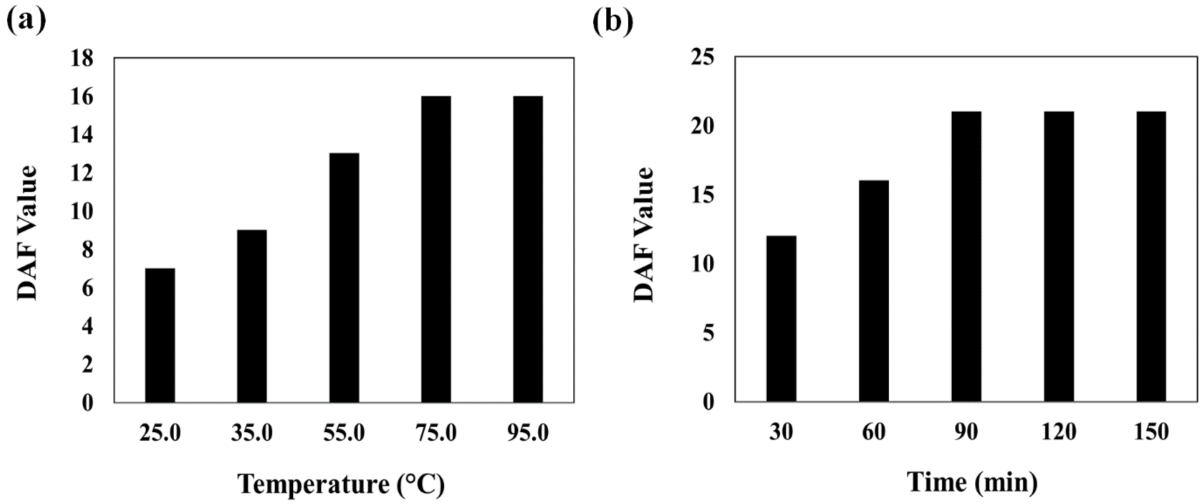

Figure S3. The effect of temperature (a) and time of interaction (b) on the sensor responses after exposed to urine samples of control and injured people.

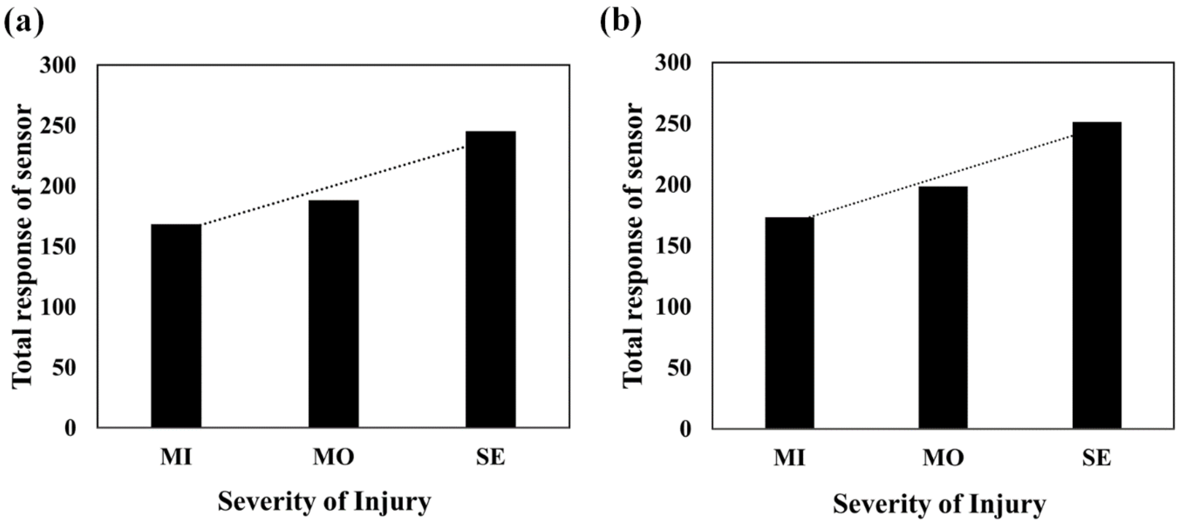

Figure S4. The correlation between the response of sensing elements S3 (a) and S7 (b) and severity of injury. The analysis was performed in oven at 75°C for 90 min.

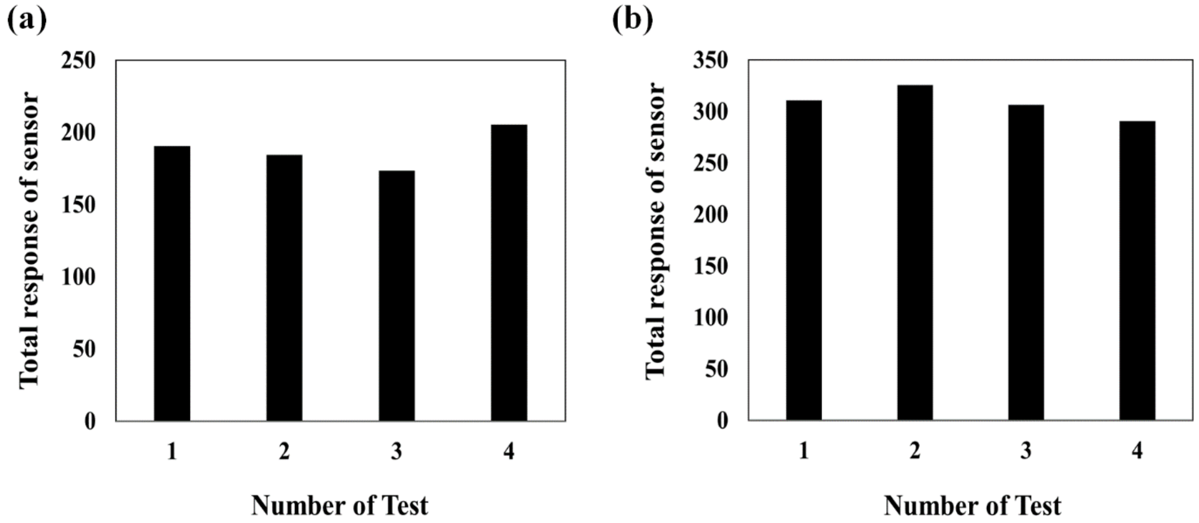

Figure S5. Evaluating the reproducibility of the sensor responses after exposing to (a) control and (b) injured samples. The analysis was performed in oven at 75°C for 90 min. The RSD % were obtained as 5.62 % and 4.67 % for control and injured samples.

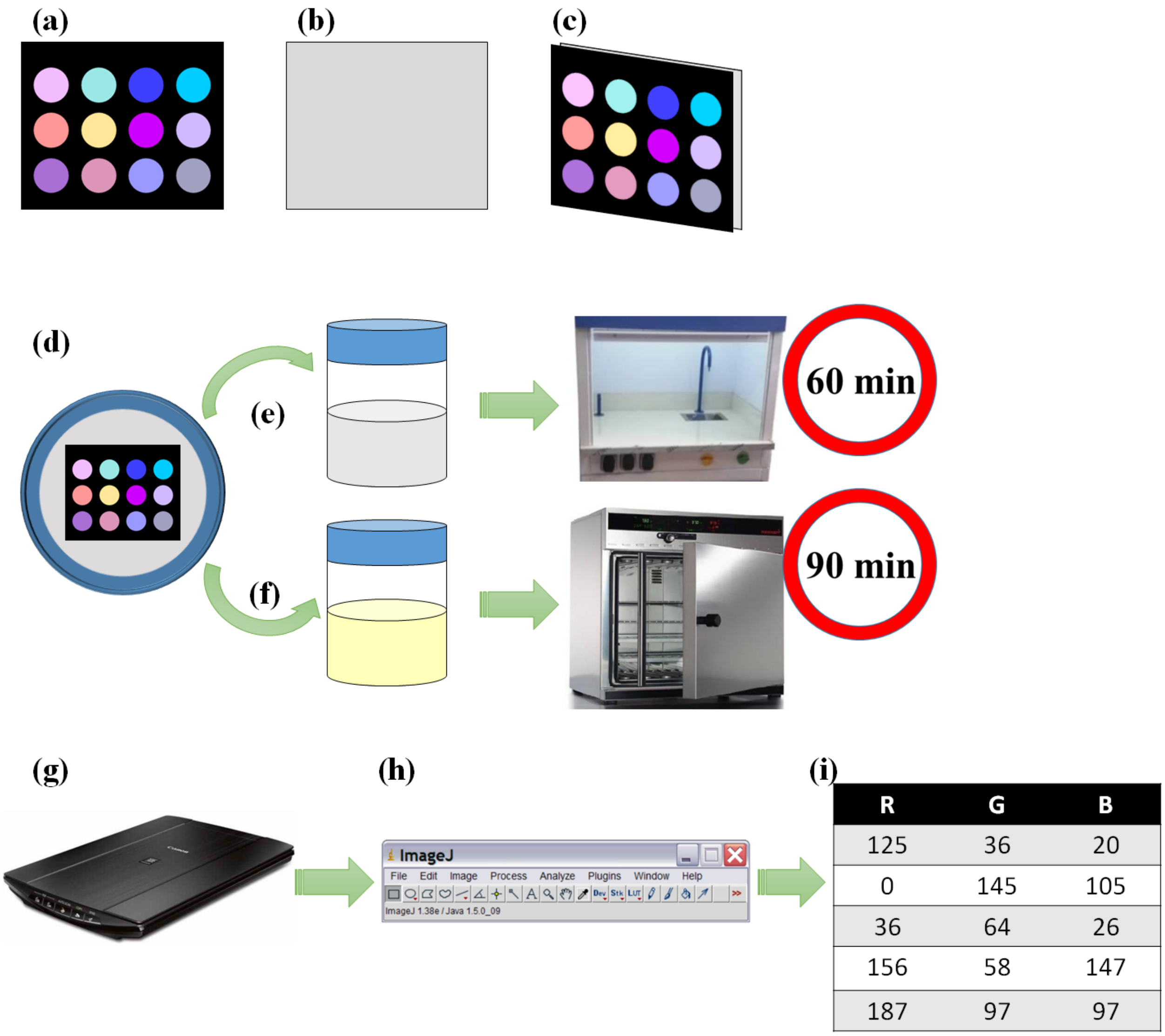

Figure S6. The schematic for the practical method: (a) the designed sensor, (b) the plastic substrate, (c) pasting the sensor on the substrate, (d) embedding the sensor to cap of the container, (e) filling the container with pure volatile compounds, transferring the container under the hood and doing the test for 60 min, (f) filling the container with urine sample, transferring the container to oven and doing the test for 90 min, (g) recording the image of sensor, (h) analysis the images with a software and (i) collecting the data for further qualitative and quantitative analysis.
